# Exploring methods for comparing the real-world effectiveness of treatments for osteoporosis: adjusted direct comparisons versus using patients as their own control

**DOI:** 10.1007/s11657-017-0375-7

**Published:** 2017-09-21

**Authors:** Linda Karlsson, Johan Mesterton, Maurille Feudjo Tepie, Michele Intorcia, Jetty Overbeek, Oskar Ström

**Affiliations:** 1 Quantify Research, Hantverkargatan 8, SE-112 21 Stockholm, Sweden; 2 Department of Learning, Informatics, Management and Ethics (LIME), Medical Management, Stockholm, Sweden; 3 Amgen UK, Uxbridge, UK; 4 Amgen (Europe) GmbH, Zug, Switzerland; 5 PHARMO Institute for Drug Outcomes Research, Utrecht, Netherlands

**Keywords:** Adjusted direct comparisons, Comparative effectiveness, Fracture incidence, Osteoporosis, Patient own control analysis, Retrospective

## Abstract

**Summary:**

Using Swedish and Dutch registry data for women initiating bisphosphonates, we evaluated two methods of comparing the real-world effectiveness of osteoporosis treatments that attempt to adjust for differences in patient baseline characteristics. Each method has advantages and disadvantages; both are potential complements to clinical trial analyses.

**Purpose:**

We evaluated methods of comparing the real-world effectiveness of osteoporosis treatments that attempt to adjust for both observed and unobserved confounding.

**Methods:**

Swedish and Dutch registry data for women initiating zoledronate or oral bisphosphonates (OBPs; alendronate/risedronate) were used; the primary outcome was fracture. In adjusted direct comparisons (ADCs), regression and matching techniques were used to account for baseline differences in known risk factors for fracture (e.g., age, previous fracture, comorbidities). In an own-control analysis (OCA), for each treatment, fracture incidence in the first 90 days following treatment initiation (the baseline risk period) was compared with fracture incidence in the 1-year period starting 91 days after treatment initiation (the treatment exposure period).

**Results:**

In total, 1196 and 149 women initiating zoledronate and 14,764 and 25,058 initiating OBPs were eligible in the Swedish and Dutch registries, respectively. Owing to the small Dutch zoledronate sample, only the Swedish data were used to compare fracture incidences between treatment groups. ADCs showed a numerically higher fracture incidence in the zoledronate than in the OBPs group (hazard ratio 1.09–1.21; not statistically significant, *p* > 0.05). For both treatment groups, OCA showed a higher fracture incidence in the baseline risk period than in the treatment exposure period, indicating a treatment effect. OCA showed a similar or greater effect in the zoledronate group compared with the OBPs group.

**Conclusions:**

ADC and OCA each possesses advantages and disadvantages. Combining both methods may provide an estimate of real-world treatment efficacy that could potentially complement clinical trial findings.

**Electronic supplementary material:**

The online version of this article (10.1007/s11657-017-0375-7) contains supplementary material, which is available to authorized users.

## Introduction

Several classes of agent are available for the prevention and treatment of osteoporosis, with the primary aim of reducing fracture risk [1]. Bisphosphonates have been the mainstay of treatment in Europe [2] and are available as oral (e.g., alendronate, risedronate and ibandronate) and intravenous (e.g., ibandronate and zoledronate) formulations. In Europe, oral bisphosphonates (OBPs) are commonly recommended as first-line treatment (most patients being prescribed alendronate), while intravenous treatments are typically reserved for second-line therapy [3]. With several osteoporosis treatment options available, it is important to develop and evaluate methods for comparing their effectiveness in real-world settings.

Bisphosphonates have been shown to reduce osteoporotic fracture risk in several randomised controlled trials (RCTs) [4–6]. Although considered the gold standard for evaluating drug safety and efficacy, RCTs often have strict inclusion and exclusion criteria, and, consequently, enrolled patients may not accurately reflect the patient population treated in clinical practice with regard to comorbidities, concomitant medication use, age or disease severity. Moreover, owing to the complexity of identifying fractures and the requirement for large sample sizes and long follow-up periods, conducting RCTs to assess the effectiveness of osteoporosis treatments is difficult and costly [7].

Retrospective observational studies using large healthcare databases may prove useful for comparing the real-world effectiveness of osteoporosis treatments [8]. While gathering such data is less costly and more time-efficient than conducting RCTs, the lack of randomisation could lead to indication bias (e.g., for treatments indicated in patients at high risk of osteoporotic fracture, the real-world patient population may be biased towards those at high fracture risk). Confounders may be known and characterised, such as age, comorbidities or previous fractures, or may be unknown or unmeasurable factors [9]. It is important to consider the possibility of confounding when interpreting data from observational studies.

Several studies have utilised administrative databases to compare the real-world effectiveness of different OBPs; Cox proportional hazard models adjusting for risk factors for fracture, including through the use of propensity score methods, have been used to estimate effect differences between OBPs [10–13] and to compare compliant vs. non-compliant patients [14]. However, these methods adjust only for known confounders. Abelson and colleagues estimated bisphosphonate effectiveness by measuring the change in fracture incidence over time and using each treatment group as its own control [9]. This method is similar to a self-controlled case series (SCCS) method in which individuals act as their own control and patient characteristics that remain constant over the observation period are accounted for [15, 16]. This method assumes that reduction in fracture incidence is not immediate following osteoporosis treatment initiation; bone mineral density (BMD) can take up to 2 years to reach its maximum level following treatment initiation [17].

No single study has used different methods for evaluating the real-world effectiveness of osteoporosis therapies that attempt to adjust for potential biases. This study evaluated two methods of comparing the real-world effectiveness of OBPs and zoledronate that attempt to adjust for observed and unobserved confounding.

## Methods

### Study sample and data sources

This study used registry data from Sweden and the Netherlands for women initiating treatment with zoledronate (intravenous injection) or OBPs (alendronate/risedronate). These antiresorptive treatments were chosen because of their differing modes of administration, which may impact on efficacy [6, 18]. Zoledronate is a second-line treatment in both Sweden and the Netherlands and may be targeted at patients at high risk of fracture. Therefore, it is an appropriate comparator to assess the ability of methods to account for baseline risk differences.

Women were included in the study if they were 50 years of age or older and initiated alendronate, risedronate or zoledronate during the identification period (1 July 2006–31 December 2011 for Sweden and 1 January 2007–30 June 2011 for the Netherlands). The index date was defined as the date of treatment initiation (date of first prescription). Women had to have available data from at least 12 months before the index date and at least 6 months after the index date. Patients were excluded if they had a diagnosis of malignancy, tumours of unknown nature or Paget’s disease, or had been prescribed the same osteoporosis treatment during the pre-index period.

Included individuals were assigned to either the zoledronate or the OBPs group. Patients could only be assigned to one group, and to maximise the size of the zoledronate group, a hierarchical approach was used such that patients initiating both OBPs and zoledronate during the identification period were assigned to the zoledronate group. Patients were followed up from treatment initiation until the first of the following: death, end of the study period (30 June 2012 for Sweden and 31 December 2011 for the Netherlands) or switch to another osteoporosis treatment (other than switching between alendronate and risedronate). Zoledronate patients with previous OBP experience were studied from the day of treatment initiation with zoledronate. No consideration was given as to whether patients terminated treatment; hence, individuals were included and followed up until censoring.

In Sweden, retrospective data from national (Swedish Prescribed Drug Register, Swedish Causes of Death Register) and regional (Stockholm regional database) registers were used [19–21], and the study was limited to the population living in Stockholm County, where zoledronate is dispensed through outpatient pharmacies and is therefore possible to identify in the national prescription register. Swedish national registers have a high degree of accuracy. The loss of patient information from the Swedish Prescribed Drug Register is less than 0.6% of all possible values, and fewer than 0.5% of all deaths are missing from the Causes of Death Register [20].

In the Netherlands, data were extracted from the PHARMO database network [22], which links drug dispensing records to hospital discharge records and other data sources using probabilistic linkage [23]; this study used the Outpatient Pharmacy Database and the Hospitalisation Database [24]. The PHARMO database covers approximately 4 million (25%) residents in the Netherlands [25].

### Covariates

Variables used to describe the treatment groups and to compare fracture incidences were age at treatment initiation and the following pre-index period variables: previous fracture; previous osteoporosis treatment (other than the index treatment); previous glucocorticoid use; filled at least one prescription of proton pump inhibitors, other gastro-protective agents or hormone replacement therapy; at least one diagnosis and/or hospitalisation for rheumatoid arthritis; at least one diagnosis and/or hospitalisation for renal insufficiency; Charlson–Quan comorbidity index (utilising the International Classification of Diseases and Related Health Problems, 10th Revision codes [26]) in patients from Sweden and Chronic Disease Score (using the Anatomical Therapeutic Chemical codes [27]) in patients from the Netherlands.

### Outcomes

The primary outcome was fracture. The Swedish data included fractures identified in hospitalised patients and those identified in an outpatient care setting, whereas the Dutch data captured only fractures identified in hospitalised patients. Multiple fractures could be included for an individual patient (baseline covariates remained unchanged for each fracture). A sensitivity analysis with first fracture as outcome was conducted.

### Analytical methods

Two methods were used to compare the real-world effectiveness of zoledronate and OBPs. Adjusted direct comparison (ADC) used Cox proportional hazard and Poisson models to compare the fracture incidences in patients treated with zoledronate and OBPs, adjusting for differences in baseline characteristics and length of follow-up between the two treatment groups. Own-control analysis (OCA) compared fracture incidences within each patient cohort over two time periods: the 90-day period immediately following (and including) treatment initiation, and the 1-year period starting 91 days after treatment initiation. In this method, each treatment group is used as its own control; the method therefore adjusts for all measured and unmeasured factors that do not change over time. OCA assumes that the onset of treatment effect is not immediate; fracture incidence in the time period immediately after treatment initiation was assumed to reflect the baseline fracture risk, and treatment effectiveness was estimated by the change in fracture incidence over time [9].

In the ADC method, crude treatment effects and effects adjusted for the baseline covariates described above were estimated. The latter effects were estimated to isolate the effect of treatment group on fracture incidence following treatment initiation. Propensity scores were calculated using logistic regression [28–30], whereby a patient’s probability of receiving a certain treatment was estimated based on their observed covariates. The estimated propensity score was used as a covariate in the Cox proportional hazard and Poisson models and in a matched-pair analysis (patients were matched 1:1 based on their score using the calliper approach). These analyses are summarised in Table 1.

**Table 1:** Summary of the analyses conducted with the ADC method

Analysis no	Survival analysis model
1	Crude Cox model
2	Crude Cox model, restricted to first fracture
3	Crude Poisson model
4	Matched-pair stratified Cox model
5	Conditional Poisson regression model
6	Cox model adjusted for covariates, restricted to first fracture
7	Cox model adjusted for covariates
8	Cox model adjusted for propensity score
9	Poisson model adjusted for covariates

*ADC* adjusted direct comparison

In the OCA method, fracture incidence in the first 90 days following treatment initiation (the baseline risk period) was assumed to reflect the untreated risk in the two treatment groups [9]. This fracture incidence was compared with the incidence in the time period that started 91 days after treatment initiation and lasted for 1 year (the treatment exposure period); a limit of 1 year was applied because fracture risk may change over time independent of treatment effectiveness [31–33]. For each treatment group, incidence rate ratios (IRRs) for the treatment exposure period vs. the baseline risk period were calculated. An IRR below 1 indicated that the fracture incidence was lower in the treatment exposure period than in the baseline risk period. To ensure that the baseline risk period reflected the risk in an untreated patient, the OCA was restricted to treatment-naïve patients in a subgroup analysis.

## Results

### Study sample and baseline characteristics

**Fig. 1:**
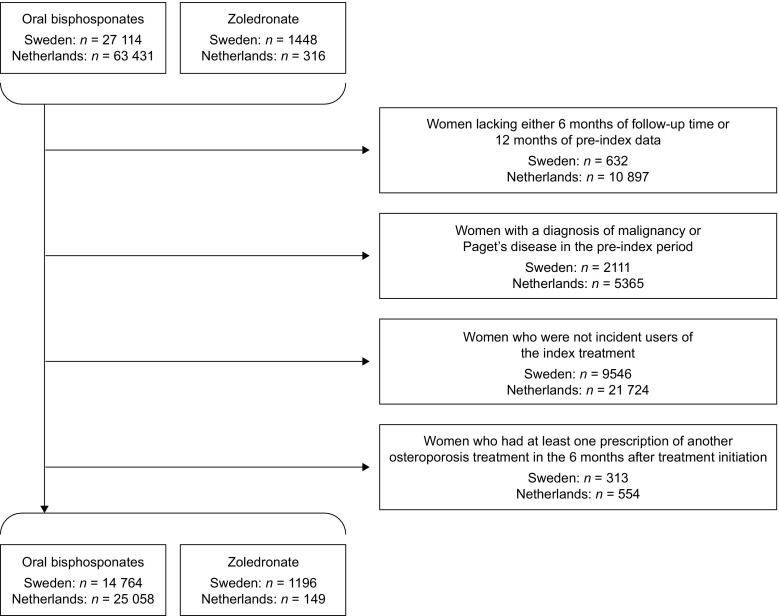
Patient selection flowchart

In total, 1196 and 149 patients receiving zoledronate and 14,764 and 25,058 receiving OBPs were eligible for inclusion in the Swedish and Dutch samples, respectively (Fig. 1). Baseline characteristics are summarised in Tables 2 and 3. Compared with patients receiving OBPs, those receiving zoledronate were more likely to have experienced a fracture during the pre-index period (24 vs. 19% in Sweden, and 9 vs. 6% in the Netherlands) and to have received previous osteoporosis treatment (52 vs. 3% in Sweden, and 43 vs. 2% in the Netherlands).

**Table 2:** Baseline characteristics for all patients included in the analyses

Characteristic	Sweden		Netherlands	
	ZOL (*n* = 1196)	OBPs (*n* = 14,764)	ZOL (*n* = 149)	OBPs (*n* = 25,058)
Age at treatment initiation, years (mean ± SD)	72 ± 9	71 ± 10	68 ± 9	71 ± 10
Previous fracture^a^	283 (24)	2832 (19)	13 (9)	1401 (6)
Previous osteoporosis treatment^b^	625 (52)	423 (3)	64 (43)	561 (2)
Previous glucocorticoid use^c^	211 (18)	3884 (26)	17 (11)	4954 (20)
Experience of PPIs^d^	463 (39)	4316 (29)	87 (58)	12,599 (50)
Experience of other gastroprotective agents^d^	49 (4)	491 (3)	11 (7)	819 (3)
Experience of HRT^d^	60 (5)	850 (6)	4 (3)	410 (2)
Rheumatoid arthritis^e^	60 (5)	896 (6)	1 (1)	101 (< 0.5)
Renal insufficiency^e^	10 (1)	107 (1)	0 (0)	56 (< 0.5)
Charlson–Quan comorbidity index (mean ± SD)^f^	0.3 ± 0.8	0.3 ± 0.7	–	–
Chronic Disease Score (mean ± SD)^f^	–	–	6 ± 4	5 ± 4

Data are presented as *n* (%) unless otherwise specified. A dash in the table indicates that the number of individuals was too small (≤ 3) to be presented. HRT hormone replacement therapy, OBP oral bisphosphonate, PPI proton pump inhibitor, SD standard deviation, ZOL zoledronate

^a^A fracture diagnosis and/or hospitalisation for a fracture in the pre-index period

^b^Experience of any osteoporosis treatment other than the index treatment in the pre-index period

^c^Filled prescriptions equivalent to ≥ 450 mg of prednisolone (corresponding to ≥ 3 months at a dose of 5 mg/day) during the pre-index period

^d^Filled at least one prescription of PPIs/gastroprotective agents/HRT in the pre-index period

^e^At least one diagnosis and/or hospitalisation for rheumatoid arthritis/renal insufficiency in the pre-index period

^f^Comorbidities were measured using the Charlson–Quan comorbidity index in Sweden and the Chronic Disease Score in the Netherlands

**Table 3:** Baseline characteristics for patients included in the analyses following propensity score matching

Characteristic	Sweden		Netherlands	
	ZOL (*n* = 974)	OBPs (*n* = 974)	ZOL (*n* = 142)	OBPs (*n* = 426)
Age at treatment initiation, years (mean ± SD)	73 ± 9	72 ± 9	68 ± 9	71 ± 9
Previous fracture^a^	179 (18)	193 (20)	12 (8)	18 (4)
Previous osteoporosis treatment^b^	403 (41)	403 (41)	57 (40)	171 (40)
Previous glucocorticoid use^c^	177 (18)	147 (15)	17 (12)	61 (14)
Experience of PPIs^d^	340 (35)	325 (33)	81 (57)	234 (55)
Experience of other gastroprotective agents^d^	36 (4)	38 (4)	10 (7)	22 (5)
Experience of HRT^d^	60 (5)	48 (5)	4 (3)	6 (1)
Rheumatoid arthritis^e^	50 (5)	39 (4)	1 (1)	3 (1)
Renal insufficiency^e^	9 (1)	4 (< 0.5)	0 (0)	0 (0)
Charlson–Quan comorbidity index (mean ± SD)^f^	0.3 ± 0.8	0.3 ± 0.7	–	–
Chronic Disease Score (mean ± SD)^f^	–	–	6 ± 4	6 ± 4

A 1-1 matching was conducted with Swedish data, whereas a 3-1 matching was conducted utilising Dutch data. Data are presented as *n* (%) unless otherwise specified. A dash in the table indicates that the number of individuals was too small (≤ 3) to be presented. HRT hormone replacement therapy, OBP oral bisphosphonate, PPI proton pump inhibitor, SD standard deviation, ZOL zoledronate

^a^A fracture diagnosis and/or hospitalisation for a fracture in the pre-index period

^b^Experience of any osteoporosis treatment other than the index treatment in the pre-index period

^c^Filled prescriptions equivalent to ≥ 450 mg of prednisolone (corresponding to ≥ 3 months at a dose of 5 mg/day) during the pre-index period

^d^Filled at least one prescription of PPIs/gastroprotective agents/HRT in the pre-index period

^e^At least one diagnosis and/or hospitalisation for rheumatoid arthritis/renal insufficiency in the pre-index period

^f^Comorbidities were measured using the Charlson–Quan comorbidity index in Sweden and the Chronic Disease Score in the Netherlands

### Fracture incidence

**Fig. 2:**
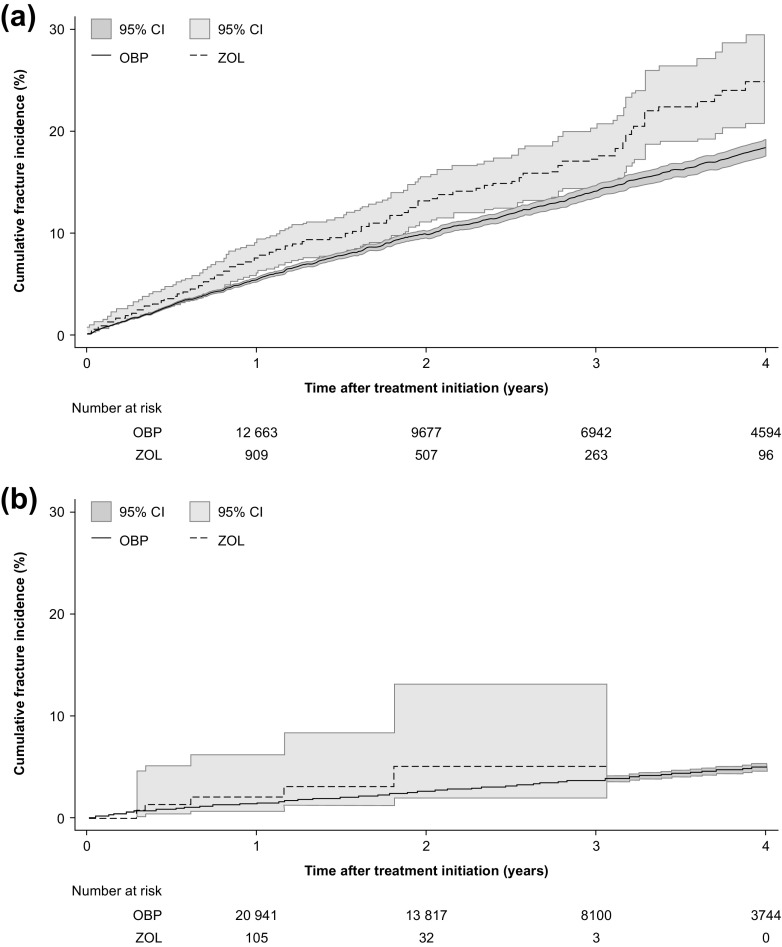
Cumulative fracture incidence in **a** Sweden and **b** the Netherlands following initiation of oral bisphosphonates and zoledronate (analysis restricted to first fracture only). Note that the Swedish fracture incidence also includes fractures identified in outpatient care. *CI* confidence interval, *OBPs* oral bisphosphonates, *ZOL* zoledronate

In the Swedish sample, the 3-year cumulative fracture incidence was high in both treatment groups (Fig. 2a). Patients receiving zoledronate had a significantly higher 3-year fracture incidence than those receiving an OBP (18 vs. 14%, respectively [log-rank test, *p* < 0.001]). Almost half of all fractures occurring after treatment initiation were identified in hospitalised patients (data not shown). The most frequent fracture types after treatment initiation were fracture of the femur including hip (22%) and fracture of the forearm (20%). This analysis was based on approximately 50,000 person-years; the OBPs group had longer mean follow-up than the zoledronate group (3.2 vs. 2.2 years, respectively).

In the Dutch sample, the 3-year cumulative fracture incidence for the OBPs group was 4% (Fig. 2b). The mean follow-up for the OBPs group was 2.4 vs. 1.5 years for the zoledronate group. The zoledronate group was too small (*n* = 149) for comparative analysis of fracture incidence; such analyses were, therefore, conducted using Swedish data only.

### Adjusted direct comparison

**Fig. 3:**
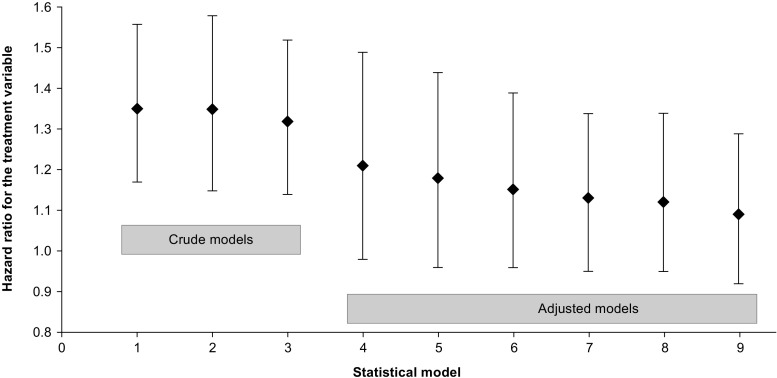
Hazard ratios based on Swedish data for the treatment covariate (defined as 1 for zoledronate and 0 for OBPs) with 95% CIs, calculated using the following models: 1—crude Cox model (*n* = 15,960), *p* value < 0.001; 2—crude Cox model, restricted to first fracture (*n* = 15,960), *p* value < 0.001; 3—crude Poisson model (*n* = 15,960), *p* value < 0.001; 4—matched-pair stratified Cox model, propensity score-matched population (*n* = 1948), *p* value 0.080; 5—conditional Poisson regression model, propensity score-matched population (*n* = 1948), *p* value 0.115; 6—Cox model adjusted for covariates, restricted to first fracture (*n* = 15,960), *p* value 0.139; 7—Cox model adjusted for covariates (*n* = 15,960), *p* value 0.161; 8—Cox model adjusted for propensity score (*n* = 15,960), *p* value 0.183; 9—Poisson model adjusted for covariates (*n* = 15,960), *p* value 0.314. *CI* confidence interval, *OBPs* oral bisphosphonates, *ZOL* zoledronate

ADC results from the Swedish data are presented in Fig. 3; hazard ratios (HRs) describe the risk of fracture for zoledronic acid vs. OBPs. The crude HRs in the survival analyses confirm the higher fracture incidence in the zoledronate group vs. the OBPs group (HR 1.32–1.35, *p* < 0.001). When controlling for baseline covariates or propensity score, or using matched-pair analysis, non-significant differences in fracture incidence were observed between the treatment groups (HR 1.09–1.21, *p* > 0.05). The results were robust when considering the first fracture only. All of the models reduced the HRs compared with the crude analyses; however, none of the models tested estimated a HR below 1.0 (i.e., a lower fracture incidence in the zoledronate than in the OBPs group).

### Own-control analysis

**Fig. 4:**
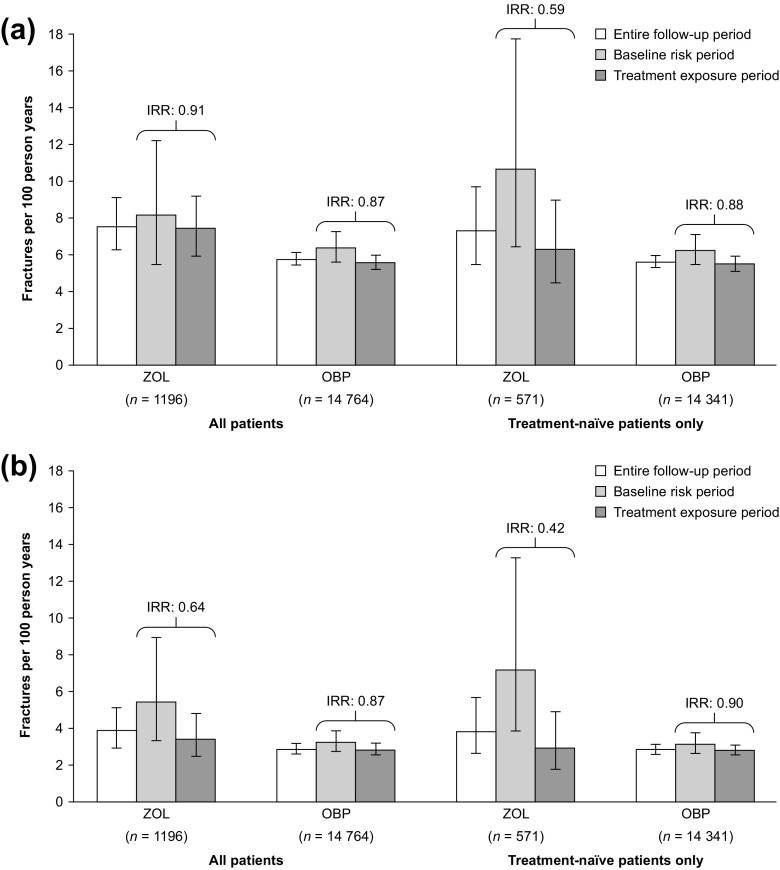
Comparison of Swedish fracture incidence and incidence rate ratios between the zoledronate and OBPs groups in three different time periods, for **a** fractures identified in hospitalised patients and outpatient care and **b** fractures identified in hospitalised patients only. IRRs were calculated as treatment exposure period vs. baseline risk period, and all patients were censored at 15 months after treatment initiation. *IRR* incidence rate ratio, *OBPs* oral bisphosphonates, *ZOL* zoledronate

OCA results from the Swedish data are presented in Fig. 4a (all fractures) and Fig. 4b (fractures identified in hospitalised patients only). Fracture incidences for the entire follow-up period are also presented. For both treatment groups, and irrespective of whether all fractures or only those identified in hospitalised patients were considered, or whether all patients or only treatment-naïve patients were considered, fracture incidence was higher in the baseline risk period than in the treatment exposure period. For all time periods (i.e., entire follow-up, baseline risk and treatment exposure periods), the fracture incidence was higher for the zoledronate group than for the OBPs group.

In the analysis of all patients and all fractures, the IRRs estimated a 9–13% lower fracture incidence in the treatment exposure period vs. the baseline risk period. When considering only fractures identified in hospitalised patients, larger differences were seen between these two time periods, especially in the zoledronate group (IRR 0.64 for only fractures identified in hospitalised patients vs. 0.91 for all fractures). For zoledronate, a larger difference in fracture incidence between these two time periods was also seen when considering only treatment-naïve patients (IRR 0.91 for all patients vs. 0.59 for treatment-naïve patients only, for all fractures); however, in this analysis, the size of the zoledronate group was reduced by more than half, and, therefore, results in the baseline risk period are based on a small number of events.

## Discussion

We have assessed different methods for comparing the effectiveness of osteoporosis treatments in women initiating zoledronate or OBPs using retrospective data from Sweden and the Netherlands. The real-world effectiveness of these treatments was also compared; this analysis was performed on data from Sweden only, owing to the small zoledronate sample size in the Netherlands. This small sample size may be the result of the PHARMO database network linking data and not including the entire population of the Netherlands, as well as the fact that zoledronate had been available for only a short time when patients were being identified for inclusion in this study.

In both countries, baseline characteristics known to influence fracture risk differed between treatment groups. In each country, more than 40% of patients initiating zoledronate had received another osteoporosis treatment in the 12 months prior to zoledronate initiation. In addition, compared with patients initiating OBPs, a higher proportion of patients initiating zoledronate had experienced a fracture in the 12 months prior to treatment initiation. These results are not surprising, considering zoledronate is a second-line treatment given to patients who have a high fracture risk, or who found administration of OBPs complex, or who were intolerant to first-line therapies [34].

The 3-year cumulative fracture incidence in the Swedish sample was estimated at 18 and 14% for the zoledronate and OBPs groups, respectively. For both treatments, this is higher than that observed in two major multinational trials investigating their efficacy (the Fracture Intervention Trial [5] and the HORIZON trial [4]). This is not surprising, however, given that Swedish fracture risks are among the highest in the world [35] and that patients in real-world clinical practice are often older and more frail than those included in RCTs [8]. In addition, several studies have shown that persistence with bisphosphonates, particularly those administered orally, is suboptimal [36–39]. In our study, no consideration was given to patients terminating treatment, so it is likely that several patients were not persistent with treatment. Given that persistence has been shown to affect anti-fracture efficacy [36, 40–43], this may also explain some of the differences between the results in our study and those in the clinical trials. Moreover, patients in trials are likely to be monitored more closely than those in observational studies, which may itself encourage persistence.

RCTs have demonstrated that zoledronate and OBPs have similar efficacies [6, 18]. However, zoledronate is commonly used at second line [34], and patients prescribed zoledronate tend to be more frail than those prescribed OBPs. It is therefore not surprising that our analysis found a higher fracture incidence in patients initiating zoledronate than in those initiating OBPs. However, given that compliance and persistence with osteoporosis treatment are associated with efficacy [44], the high persistence seen with yearly IV zoledronate infusions [45], which guarantees 100% persistence and compliance for at least 12 months, may be expected to result in improved effectiveness compared with OBPs, which require frequent self-administration and have lower persistence and compliance. However, it should be noted that factors other than good compliance and persistence also play a role in real-world effectiveness.

The crude HRs for fracture incidence for the zoledronate group relative to the OBPs group ranged from 1.32 to 1.35 (*p* < 0.001). When adjusting for baseline covariates (ADC method), and using different models and propensity score adjustments, this was reduced to a non-significant estimate of approximately 1.10 (range 1.09–1.21, *p* > 0.05). This implies that, while the data registries used in our study included a number of known risk factors for fracture, information on additional potential risk factors (e.g., BMD, smoking, body mass index, fall propensity and socioeconomic variables) may be needed to successfully adjust for differences in baseline fracture risk. Information on several potential risk factors could be obtained from electronic medical records or hospital databases, enabling further retrospective research on the extent to which the differences in baseline risk can be accounted for.

OCA is the only conceivable method whereby the patient group acts as its own control, making it unnecessary to capture all the relevant risk factors for a fracture. The results from the OCA showed that the incidence of any fracture was lower in the treatment exposure period than in the baseline risk period. When including all fractures in all patients, the fracture incidence reductions were similar in the two treatment groups. However, in the analysis of fractures in hospitalised patients only and in the subgroup analysis of treatment-naïve patients, there was a trend towards a greater reduction in fracture incidence in the zoledronate than in the OBPs group. In the OBPs group, results remained unchanged when considering only fractures in hospitalised patients compared with the primary analysis which included fractures identified in both the hospital and outpatient settings. The reason for this pattern is unknown and warrants further study.

For zoledronate, the IRRs estimated using the OCA method were similar to those reported by Black and colleagues [4] for zoledronate vs. placebo (relative risk 0.67) and by Cummings and colleagues [5] for alendronate vs. placebo (relative risk 0.86). Our estimates for OBPs were slightly higher than those reported by Abelson and colleagues (IRR 0.72 and 0.79 for alendronate and risedronate, respectively) [9]. When considering only treatment-naïve patients, the IRR decreased from 0.91 to 0.59 in the zoledronate group. This is likely because over 50% of zoledronate-treated patients had previously received an OBP and benefited from some fracture protection and thus had a reduced fracture risk at baseline compared with those patients who were OBP-treatment-naïve. With zoledronate used as a second-line treatment in Sweden, it may be more appropriate to compare only treatment-naïve patients in the two treatment groups in this study, even though the sample size of the zoledronate group is notably smaller than that of the OBPs group.

In this study, time of onset of treatment effect was assumed to be 90 days. The exact time of onset is not known and may vary between different osteoporosis treatments; this is a factor to which the OCA method is sensitive, by definition. While more research is needed to identify the time to onset of treatment effect for different antiresorptive treatments, it is unlikely that onset of effect occurs at a specific time point (e.g., 90 days). Consequently, this type of approach should be used only to estimate comparative effectiveness, rather than effectiveness of individual treatments. Furthermore, it should be noted that in many cases, treatment is initiated because of a fracture. In such cases, the elevated risk of subsequent fracture in the period immediately after a fracture may be related to this first fracture rather than to a high long-term fracture risk. The differences between the two patient groups in fracture risk at treatment initiation could also affect the possible size of the treatment effect and limit comparisons between treatments [9]. It should also be noted that the treatment exposure period was limited to 1 year to take into account the possibility that a patient’s fracture risk may change over time independently of treatment effectiveness [31–33]. Hence, this approach considers only a limited time period and does not investigate the long-term effectiveness of the different treatments. This approach does not account for variables that change over time, such as concomitant medications and comorbidities.

The strength of both methods used to assess effectiveness is that real-world persistence is accounted for. However, studies suggest persistence differs between patients receiving zoledronate and those receiving OBPs; this was not adjusted for in our study [39]. Compared with RCTs, the less intensive monitoring of patients in real-world clinical practice could be expected to accentuate differences in persistence/compliance profiles across treatments. A limitation of this study is the relatively small sample of patients receiving zoledronate, which made it hard to draw definite conclusions about the analytical approaches explored.

## Conclusion

The present exploration of methods for assessing real-world effectiveness provides useful information regarding the challenges in estimating the real-world effectiveness of osteoporosis treatments.

The Swedish and Dutch retrospective data sources used appear suitable for obtaining robust estimates of fracture incidence. Given that the efficacy of zoledronate has been demonstrated to be similar to or higher than that of OBPs in RCTs, any failure to show that zoledronate is as effective as OBPs in clinical practice is likely a result of unobserved differences in patient characteristics (e.g., BMD), particularly when adopting an ADC approach which does not account for unmeasured confounders. The ADC between these treatments was deemed not to account sufficiently for differences in underlying fracture risk at treatment initiation. While we found the method whereby patients acted as their own controls to have potential because it adjusts for both measured and unmeasured confounders, it does not account for factors that change over time, and the necessarily short baseline risk period (only 90 days) meant that large patient samples would be needed to accurately estimate risk of fracture in this time window.

Overall, owing to the methodological challenges involved in estimating real-world and comparative effectiveness, these types of analyses should be regarded as a potential complement to treatment efficacy observed in RCTs. Each method has advantages and disadvantages; so, it may therefore be advisable to consider using both approaches in order to provide a broad overview of treatment efficacy in real-world practice.

## Electronic supplementary material

ESM 1 (DOCX 30 kb)

## References

[1] Kanis JA (2013). European guidance for the diagnosis and management of osteoporosis in postmenopausal women. Osteoporos Int.

[2] Strom O (2011). Osteoporosis: burden, health care provision and opportunities in the EU: a report prepared in collaboration with the International Osteoporosis Foundation (IOF) and the European Federation of Pharmaceutical Industry Associations (EFPIA). Arch Osteoporos.

[3] Hernlund E (2013). Osteoporosis in the European Union: medical management, epidemiology and economic burden. A report prepared in collaboration with the International Osteoporosis Foundation (IOF) and the European Federation of Pharmaceutical Industry Associations (EFPIA). Arch Osteoporos.

[4] Black DM (2007). Once-yearly zoledronic acid for treatment of postmenopausal osteoporosis. N Engl J Med.

[5] Cummings SR (1998). Effect of alendronate on risk of fracture in women with low bone density but without vertebral fractures: results from the Fracture Intervention Trial. JAMA.

[6] Jansen JP (2011). The efficacy of bisphosphonates in the prevention of vertebral, hip, and nonvertebral-nonhip fractures in osteoporosis: a network meta-analysis. Semin Arthritis Rheum.

[7] Kanis JA (2002). Uncertain future of trials in osteoporosis. Osteoporos Int.

[8] Lindsay BD (2007). Beyond clinical trials: the importance of large databases in evaluating differences in the effectiveness of bisphosphonate therapy in postmenopausal osteoporosis. Bone.

[9] Abelson A (2010). Longitudinal change in clinical fracture incidence after initiation of bisphosphonates. Osteoporos Int.

[10] Cadarette SM (2008). Relative effectiveness of osteoporosis drugs for preventing nonvertebral fracture. Ann Intern Med.

[11] Curtis JR (2009). RisedronatE and ALendronate Intervention over Three Years (REALITY): minimal differences in fracture risk reduction. Osteoporos Int.

[12] Harris ST (2009). Risk of fracture in women treated with monthly oral ibandronate or weekly bisphosphonates: the eValuation of IBandronate Efficacy (VIBE) database fracture study. Bone.

[13] Silverman SL (2007). Effectiveness of bisphosphonates on nonvertebral and hip fractures in the first year of therapy: the risedronate and alendronate (REAL) cohort study. Osteoporos Int.

[14] Lakatos P (2014). Comparative statistical analysis of osteoporosis treatment based on Hungarian claims data and interpretation of the results in respect to cost-effectiveness. Osteoporos Int.

[15] Farrington CP (1995). Relative incidence estimation from case series for vaccine safety evaluation. Biometrics.

[16] Maclure M (1991). The case-crossover design: a method for studying transient effects on the risk of acute events. Am J Epidemiol.

[17] Bonnick S et al (2006) Comparison of weekly treatment of postmenopausal osteoporosis with alendronate versus risedronate over two years. J Clin Endocrinol Metab 91(7):2631–2637 10.1210/jc.2005-2602 16636120

[18] Serrano AJ (2013). Systematic review and meta-analysis of the efficacy and safety of alendronate and zoledronate for the treatment of postmenopausal osteoporosis. Gynecol Endocrinol.

[19] Stockholm County Council. [Accessed 2015 3 February]; Available from: http://www.gups.sll.se/val/Valhandbok_Kortversion.pdf

[20] The National Board of Health and Welfare. [Accessed 2017 June 16]; Available from: http://www.socialstyrelsen.se

[21] Wettermark B (2007). The new Swedish Prescribed Drug Register—opportunities for pharmacoepidemiological research and experience from the first six months. Pharmacoepidemiol Drug Saf.

[22] Herings RMC, Pedersen L (2012) Pharmacy-based medical record linkage systems. In: Strom BL, Kimmel SE, Hennessy S (eds) Pharmacoepidemiology, 5th edn. Wiley-Blackwell, Oxford. 10.1002/9781119959946.ch18

[23] Herings R, Pedersen L (2012) Pharmacy-based medical record linkage systems, in Pharmacoepidemiology, Fifth edition. In: B.L. Strom, S.E. Kimmel, and S. Hennessy (Eds) p. 270–286

[24] Meijer WM (2008). Relationship between duration of compliant bisphosphonate use and the risk of osteoporotic fractures. Curr Med Res Opin.

[25] Pharmo database network [Accessed 2017 June 16]; Available from: http://pharmo.nl/what-we-have/pharmo-database-network

[26] Quan H (2005). Coding algorithms for defining comorbidities in ICD-9-CM and ICD-10 administrative data. Med Care.

[27] Von Korff M, Wagner EH, Saunders K (1992). A chronic disease score from automated pharmacy data. J Clin Epidemiol.

[28] D'Agostino RB (1998). Propensity score methods for bias reduction in the comparison of a treatment to a non-randomized control group. Stat Med.

[29] Imbens GW (2000). The role of the propensity score in estimating dose-response functions. Biometrika.

[30] Rosenbaum PR, Rubin DR (1983). The central role of the propensity score in observational studies for causal effects. Biometrika.

[31] Ahmed LA (2013). Progressively increasing fracture risk with advancing age after initial incident fragility fracture: the Tromso study. J Bone Miner Res.

[32] Johnell O (2004). Fracture risk following an osteoporotic fracture. Osteoporos Int.

[33] Riggs BL (2006). Population-based analysis of the relationship of whole bone strength indices and fall-related loads to age- and sex-specific patterns of hip and wrist fractures. J Bone Miner Res.

[34] The National Board of Health and Welfare. Nationella riktlinjer för rörelseorganens sjukdomar 2012 [Accessed 2015 9 September]; Available from: http://www.socialstyrelsen.se/Lists/Artikelkatalog/Attachments/18665/2012-5-1.pdf

[35] Dhanwal DK (2011). Epidemiology of hip fracture: worldwide geographic variation. Indian J Orthop.

[36] Hadji P (2012). GRAND: the German retrospective cohort analysis on compliance and persistence and the associated risk of fractures in osteoporotic women treated with oral bisphosphonates. Osteoporos Int.

[37] Hadji P (2016). GRAND-4: the German retrospective analysis of long-term persistence in women with osteoporosis treated with bisphosphonates or denosumab. Osteoporos Int.

[38] Karlsson L (2015). Persistence with denosumab and persistence with oral bisphosphonates for the treatment of postmenopausal osteoporosis: a retrospective, observational study, and a meta-analysis. Osteoporos Int.

[39] Lakatos P (2016). A retrospective longitudinal database study of persistence and compliance with treatment of osteoporosis in Hungary. Calcif Tissue Int.

[40] Hoer A (2009). Influence on persistence and adherence with oral bisphosphonates on fracture rates in osteoporosis. Patient Prefer Adherence.

[41] Huybrechts KF, Ishak KJ, Caro JJ (2006). Assessment of compliance with osteoporosis treatment and its consequences in a managed care population. Bone.

[42] Lakatos P et al (2013) Compliance protects against fracture in women with postmenopausal osteoporosis in Hungary. Value Health 16(7):A567

[43] Siris ES (2009). Impact of osteoporosis treatment adherence on fracture rates in North America and Europe. Am J Med.

[44] Cramer JA (2008). Medication compliance and persistence: terminology and definitions. Value Health.

[45] Ziller V (2012). Persistence and compliance of medications used in the treatment of osteoporosis—analysis using a large scale, representative, longitudinal German database. Int J Clin Pharmacol Ther.

