# Supplementary material for: Exploring methods for comparing the real-world effectiveness of treatments for osteoporosis: adjusted direct comparisons versus using patients as their own control
Source: Arch Osteoporos. 2017 Sep 21;12(1):81. doi: 10.1007/s11657-017-0375-7 (PMC5608859; doi:10.1007/s11657-017-0375-7)
Supplement: Supplementary file 1 — (DOCX 30 kb) [file 11657_2017_375_MOESM1_ESM.docx]

Supplemental file: Fracture, diagnosis and treatment codes

S1. Fracture codes

The fracture codes used in the study were chosen to be similar to those included in clinical trials. Fractures to the skull, face, foot, mandible, fingers, metacarpals are not associated with decreased bone mineral density and were excluded**.**

The effectiveness of osteoporosis treatments is usually measured for low energy fractures only, where the fracture is caused by a fall in the same plane (e.g. standing or from a chair), and the accident causing the fracture can be recorded in ICD-10 (W0.x). While over 90% of hospitalized spine fractures and 95% of hip fractures are caused by low energy trauma this information is often not recorded, making it difficult to distinguish fractures caused by high energy trauma^[[1]](#footnote-1)^.

**Table 1** Included fracture codes

| **Fracture type** | **ICD-10** | **Notes** |
| --- | --- | --- |
| Neck | S12.x |  |
| Rib, sternum, thoracic spine | S22.x |  |
| Lumbar spine, pelvis | S32.x |  |
| Shoulder, upper arm | S42.x |  |
| Forearm | S52.x |  |
| Wrist and hand | S62.0x, S62.8x | Fractures to the fingers and metacarpals were excluded |
| Femur (including hip) | S72.x |  |
| Lower leg (including ankle) | S82.x |  |
| Multiple body regions | T02.1x - T02.9x | Multiple fractures engaging the skull were excluded |
| Spine, unspecified | T08.x |  |
| Upper limb, unspecified | T10.x |  |
| Lower limb, unspecified | T12.x |  |
| Unspecified osteoporotic fracture | M80.x |  |

S2. Diagnosis codes

**Table 2** Codes for exclusion criteria

| **Diagnosis** | **ICD-10** |
| --- | --- |
| Malignancies^1^ | C.0-C43.x, C45.x-C.97.x |
| Tumours of unknown nature | D37.x-D48.x |
| Paget’s disease | M88.x |

^1^Basal cell cancers (c.44.x) are usually not harmful and were therefore not excluded

**Table 3** Comorbid condition codes

| **Condition** | **ICD-10** | **Charlson Weight** |
| --- | --- | --- |
| Myocardial infarction | I21.x | 1 |
|  | I22.x |  |
|  | I25.2 |  |
| Congestive heart failure | I09.9 | 1 |
|  | I11.0 |  |
|  | I13.0 |  |
|  | I13.2 |  |
|  | I25.5 |  |
|  | I42.0 |  |
|  | I42.5‑I42.9 |  |
|  | I43.x |  |
|  | I50.x |  |
|  | P29.0 |  |
| Peripheral vascular disease | I70.x | 1 |
|  | I71.x |  |
|  | I73.1 |  |
|  | I73.8 |  |
|  | I73.9 |  |
|  | I77.1 |  |
|  | I79.0 |  |
|  | I79.2 |  |
|  | K55.1 |  |
|  | K55.8 |  |
|  | K55.9 |  |
|  | Z95.8 |  |
|  | Z95.9 |  |
| Cerebrovascular disease | G45.x | 1 |
|  | G46.x |  |
|  | H34.0 |  |
|  | I60.x-I69.x |  |
| Dementia | F00.x-F03.x | 1 |
|  | F05.1 |  |
|  | G30.x |  |
|  | G31.1 |  |
| Chronic pulmonary disease | I27.8 | 1 |
|  | I27.9 |  |
|  | J40.x-J.47.x |  |
|  | J60.x-J67.x |  |
|  | J68.4 |  |
|  | J70.1 |  |
|  | J70.3 |  |
| Rheumatic disease | M05.x | 1 |
|  | M06.x |  |
|  | M31.5 |  |
|  | M32.x-M34.x |  |
|  | M35.1 |  |
|  | M35.3 |  |
|  | M36.0 |  |
| Peptic ulcer disease | K25.x-K28.x | 1 |
| Mild liver disease | B18.x | 1 |
|  | K70.0-K70.3 |  |
|  | K70.9 |  |
|  | K71.3–K71.5 |  |
|  | K71.7 |  |
|  | K73.x |  |
|  | K74.x |  |
|  | K76.0 |  |
|  | K76.2–K76.4 |  |
|  | K76.8 |  |
|  | K76.9 |  |
|  | Z94.4 |  |
| Diabetes without chronic complication | E10.0 | 1 |
|  | E10.1 |  |
|  | E10.6 |  |
|  | E10.8 |  |
|  | E10.9 |  |
|  | E11.0 |  |
|  | E11.1 |  |
|  | E11.6 |  |
|  | E11.8 |  |
|  | E11.9 |  |
|  | E12.0 |  |
|  | E12.1 |  |
|  | E12.6 |  |
|  | E12.8 |  |
|  | E12.9 |  |
|  | E13.0 |  |
|  | E13.1 |  |
|  | E13.6 |  |
|  | E13.8 |  |
|  | E13.9 |  |
|  | E14.0 |  |
|  | E14.1 |  |
|  | E14.6 |  |
|  | E14.8 |  |
|  | E14.9 |  |
| Diabetes with chronic complication | E10.2–E10.5 | 2 |
|  | E10.7 |  |
|  | E11.2–E11.5 |  |
|  | E11.7 |  |
|  | E12.2–E12.5 |  |
|  | E12.7 |  |
|  | E13.2–E13.5 |  |
|  | E13.7 |  |
|  | E14.2–E14.5 |  |
|  | E14.7 |  |
| Hemiplegia or Paraplegia | G04.1 | 2 |
|  | G11.4 |  |
|  | G80.1 |  |
|  | G80.2 |  |
|  | G81.x |  |
|  | G82.x |  |
|  | G83.0–G83.4 |  |
|  | G83.9 |  |
| Renal disease | I12.0 | 2 |
|  | I13.1 |  |
|  | N03.2–N03.7 |  |
|  | N05.2–N05.7 |  |
|  | N18.x |  |
|  | N19.x |  |
|  | N25.0 |  |
|  | Z49.0–Z49.2 |  |
|  | Z94.0 |  |
|  | Z99.2 |  |
| Malignancy | C00.x–C26.x | 2 |
|  | C30.x–C34.x |  |
|  | C37.x–C41.x |  |
|  | C43.x |  |
|  | C45.x–C58.x |  |
|  | C60.x–C76.x |  |
|  | C81.x–C85.x |  |
|  | C88.x |  |
|  | C90.x–C97.x |  |
| Moderate to severe liver disease | I85.0 | 3 |
|  | I85.9 |  |
|  | I86.4 |  |
|  | I98.2 |  |
|  | K70.4 |  |
|  | K71.1 |  |
|  | K72.1 |  |
|  | K72.9 |  |
|  | K76.5 |  |
|  | K76.6 |  |
|  | K76.7 |  |
| Metastatic solid tumor | C77.x–C80.x | 6 |
| HIV/AIDS | B20.x–B22.x | 6 |
|  | B24.x |  |

**Table 4** Rheumatoid arthritis codes

| **Condition** | **ICD-10** |
| --- | --- |
| Rheumatoid arthritis with engagement of organs and organ systems | M053.x |
| Rheumatoid arthritis, specified location | M058.x |
| Seropositive Rheumatoid arthritis | M059.x |
| Seronegative Rheumatoid arthritis | M069.x |
| Other specified Rheumatoid arthritis | M08.x |
| Rheumatoid arthritis, unspecified | M069.x |

**Table 5** Renal insufficiency codes

| **Condition** | **ICD-10** |
| --- | --- |
| Renal insufficiency | N17.x, N18.x, N19.x |

S3. Treatment codes

The following tables show the treatments used to study patient characteristics or fracture outcomes (“studied treatments”), treatments used to define osteoporosis treatment experience, and glucocorticoid use and gastroprotective agents.

**Table 6** ATC codes for studied treatments

| **Studied treatments** | **ATC** | **Notes** |
| --- | --- | --- |
| Alendronate | M05BA04 | OW^1^ or OD^2^ |
| Alendronate + kolekalciferol | M05BB03 | Combination, OW^1^ |
| Ibandronate | M05BA06 | Oral OM^4^ and IV 3M^5^ have the same ATC |
| Risedronate | M05BA07 | OW^1^ or OD^2^ |
| Risedronate + calcium | M05BB02 | Combination, OW^1^ |
| Risedronate + calcium + kolekalciferol | M05BB04 | Combination. OW^1^ |
| Zoledronate | M05BA08 | Aclasta, I.v. infusion 12M^6^ |

**Table 7** ATC codes to categorise a patient as treatment experienced or not

| **Treatment experience** | **ATC** | **Notes** |
| --- | --- | --- |
| Alendronate | M05BA04 | OW^1^ or OD^2^ |
| Alendronate + kolekalciferol | M05BB03 | Combination, OW^1^ |
| Denosumab | M05BX04 | S.c. injection 6M^3^ |
| Ibandronate | M05BA06 | Oral OM^4^ and IV 3M^5^ have the same ATC |
| Risedronate | M05BA07 | OW^1^ or OD^2^ |
| Risedronate + calcium | M05BB02 | Combination, OW^1^ |
| Risedronate + calcium + kolekalciferol | M05BB04 | Combination. OW^1^ |
| Zoledronate | M05BA08 | I.v. infusion 12M^6^ |
| Etidronate | M05BA01 | Cyclical 14 OD^2^ every 3M^5^ |
| Etidronate + calcium | M05BB01 | Combination, cyclical 14 OD^2^ every 3M^5^, |
| Strontium Ranelate | M05BX03 | OD^2^ |
| Raloxifene | G03XC01 | OD^2^ |
| Bazedoxifene | G03XC02 | OD^2^ |
| Teriparatide | H05AA02 | S.c. injection, OD^2^ |
| Parathyroid hormone 1-84 | H05AA03 | S.c. injection, OD^2^ |
| Hormone Replacement Therapy | G03C.x, G03F.x |  |

**Table 8** ATC codes for glucocorticoid use and gastroprotective agents

|  | **ATC** |
| --- | --- |
| Glucocorticoid use |  |
| Oral glucocorticoids | H02AB.x |
| Gastroprotective agents |  |
| Proton Pump Inhibitors | A02BC.x, A02BD.x |
| Other Gastroprotective agents | A02BA.x, A02BB.x, A02BX02, A02BX13 |

^1^Once weekly, ^2^Once daily, ^3^Every 6 months, ^4^Once monthly, ^5^Every three months, ^6^Every 12 months

**Table 9** Filled prescriptions of glucocorticoids (ATC: H02AB.x) equivalent to ≥450 mg of prednisolone (corresponding to ≥3 months at a dose of 5 mg/day)

| Glucocorticoid | Definition of glucoco**r**ticoid use in the pre-index period (≥ x mg during the pre-index period) |
| --- | --- |
| Cortisone | 2,250 |
| Hydrocortisone | 1,800 |
| Methylprednisolone | 360 |
| Paramethasone | 180 |
| Prednisolone | 450 |
| Prednisone | 450 |
| Triamcinolone | 360 |
| Betamethasone | 67.5 |
| Dexamethasone | 67.5 |

1. Johnell, O., et al., *The burden of hospitalised fractures in Sweden.* Osteoporos Int, 2005. **16**(2): p. 222-8. [↑](#footnote-ref-1)
